# Global gene expression profiling identifies ALDH2, CCNE1 and SMAD3 as potential prognostic markers in upper tract urothelial carcinoma

**DOI:** 10.1186/1471-2407-14-836

**Published:** 2014-11-18

**Authors:** Song Wu, Jiahao Chen, Pei Dong, Shiqiang Zhang, Yingying He, Liang Sun, Jialou Zhu, Yanbing Cheng, Xianxin Li, Aifa Tang, Yi Huang, Yaoting Gui, Chunxiao Liu, Guosheng Yang, Fangjian Zhou, Zhiming Cai, Rongfu Wang

**Affiliations:** Institute of Immunology, Zhongshan School of Medicine, Sun Yat-sen University, Guangzhou, Guangdong 510060 China; Shenzhen Second People’s Hospital, The First Affiliated Hospital of Shenzhen University, Shenzhen, Guangdong 518035 China; Department of Urology, Sun Yat-Sen University Cancer Center, Guangzhou, Guangdong 510060 China; National-regional Key Technology Engineering Laboratory for Clinical Application of Cancer Genomics, Shenzhen Key Laboratory of Genitourinary Tumor, Shenzhen, Guangdong 518036 China; BGI-Shenzhen, Shenzhen, Guangdong 518083 China; Guangdong and Shenzhen Key Laboratory of Male Reproductive Medicine and Genetics, Institute of Urology, Peking University Shenzhen Hospital, Shenzhen PKU-HKUST Medical Center, Shenzhen, Guangdong 518036 China; Department of Urology, Zhujiang Hospital, Southern Medical University, Guangzhou, 510282 China; Department of Urology, Guangzhou Second People’s Hospital, Guangzhou, Guangdong 510282 China; Department of Cell Biology, Albert Einstein College of Medicine, Bronx, NY 10461 USA

**Keywords:** Upper tract urothelial carcinoma of renal pelvis, Global gene expression profiling, ALDH2, CCNE1, SMAD3, Prognosis

## Abstract

**Background:**

Current knowledge about the molecular properties and prognostic markers of upper tract urothelial carcinoma (UTUC) is sparse and often based on bladder urothelial carcinoma (UC), which is thought to share common risk factors with UTUC. However, studies have suggested that differences exist regarding tumor behavior and molecular biology of these cancers, comprehensive investigations are needed to guide the clinical management of UTUC. In recent years, massively parallel sequencing has allowed insights into the biology of many cancers, and molecular prognostic markers based on this approach are rapidly emerging. The goal of this study was to characterize the gene expression patterns of UTUC using massively parallel sequencing, and identify potential molecular markers for prognosis in patients with UTUC.

**Methods:**

We compared the genome-wide mRNA expression profile of cancer and matched normal tissues from 10 patients with UTUC to identify significantly deregulated genes. We also examined the protein levels of prognostic marker candidates in 103 patients with UTUC, and tested the association of these markers with overall survival using Kaplan-Meier model and Cox regression.

**Results:**

Functional enrichment of significantly deregulated genes revealed that expression patterns of UTUC were characterized by disorders of cell proliferation and metabolism. And we also compared the expression profile of UTUC with that of bladder UC. Our results highlighted both shared (e.g. disorders of cell cycling and growth signal transduction) and tumor-specific (e.g. abnormal metabolism in UTUC and disruptions of adhesion pathways in bladder UC) features of these two cancers. Importantly, we identified that low protein expression of ALDH2 while high CCNE1 and SMAD3 were significantly associated with increased depth (*P <0.05) and lower overall survival (***P <0.0001) in an independent set of 103 patients. Multivariate Cox regression revealed that all these three genes were independent prognostic indicators in patients with UTUC (***P <0.001).

**Conclusions:**

In conclusion, our study characterized the comprehensive expression profile of UTUC and highlighted both commons and differences in expression patterns between UTUC and bladder UC. And we, for the first time, revealed that ALDH2, CCNE1 and SMAD3 are associated with prognosis in patients with UTUC.

**Electronic supplementary material:**

The online version of this article (doi:10.1186/1471-2407-14-836) contains supplementary material, which is available to authorized users.

## Background

UTUC of renal pelvis is relatively rare, but aggressive type of kidney cancer with high recurrence rates. It comprises of ~8.4% of histologically confirmed cancers in kidney and approximately 5% of all urothelial neoplasms [1, 2]. Current knowledge about the molecular basis of UTUC is sparse and often based on bladder UC, which is the predominant subtype of UC and thought to share common risk factors with UTUC like cigarette smoking and use of phenacetin-containing analgesics [2, 3]. However, studies have suggested that differences exist regarding tumor location and behavior between the upper and the lower urinary tract [4–6]. In addition, Catto et al. showed that distinct patterns of microsatellite instability and promoter methylation occur in these cancers [7, 8], comprehensive studies therefore are needed to guide the clinical managements of UTUC. Until recently, most of the efforts for identifying prognostic indicators focused on only a few pre-selected genes, tumor stage and grade still represent the best-established prognostic indicators in patients with UTUC [3, 4]. It is of paramount importance to increase our understanding of the molecular basis like disrupted pathways of this cancer to refine the clinical decision-making process. In recent years, massive expression profiling techniques such as microarray and next-generation sequencing has allowed comprehensive insights into both the biology and clinical aspect of many cancers, and molecular prognostic markers based on this approach are rapidly emerging [9–11]. Compared to microarrays, sequence-based profiling does not suffer from cross-hybridization of mRNA sequences and has higher reproducibility, and it can achieves the measurement of gene expression level with unlimited dynamic range [12].

Here, using massively parallel sequencing, we compared the expression patterns of UTUCs and matched normal controls aiming to characterize the mRNAs spectra as well as identify potential molecular prognostic markers of this cancer. We identified that the expression patterns of UTUC were characterized by disorders of cell proliferation and metabolism. And we revealed that UTUC and bladder UC shared common molecular features (e.g. disorders of cell cycling and growth signal transduction), while they also have tumor-specific features (e.g. abnormal metabolism in UTUC and disruptions of adhesion pathways in bladder UC). Importantly, we identified that low protein expression of ALDH2 while high CCNE1 and SMAD3 were novel independent predictors of adverse outcome in patients with UTUC.

## Methods

### Sample collection

Written informed consents were obtained from all the 10 patients with UTUC of renal pelvis, and this study was approved by the institutional review board of Sun Yat-sen University (Guangzhou, China). None of the patients in this study underwent radiotherapy or chemotherapy before surgery. Histological examination and clinical diagnosis of the tumorous and normal adjacent tissues from renal pelvis in patients were performed by the Cancer Center of Sun Yat-sen University. Fresh tissues were immediately immersed in RNAlater (Qiagen; Germany) after surgical resection and stored at 4°C overnight to allow thorough penetration of the tissues, which were thereafter stored at -80°C. Hematoxylin-eosin (HE) staining were performed to examine the percentage of tumor cells, and tumor tissues containing more than 80% tumor cells were selected for further investigation. We also confirmed using histopathologic examination that the normal tissue did not contain any cancer cells. The disease stage of each patient was classified according to the 2002 American Joint Committee on Cancer (AJCC) staging system. Information on all these patients is summarized in Additional file 1: Table S1.

### Gene expression profiling using digital gene expression sequencing

Library construction of digital gene expression sequencing (DGE; BGI-Shenzhen, China) generates tags with 21 base pairs (bp) from the 3’ ends of each transcript, and such tags are utilized to represent the expression levels of transcripts [12]. Sequencing libraries were prepared as before [13]. In brief, after extraction of total RNA, we synthesized double-stranded cDNA from RNA using oligo (dT)_18_ beads (Invitrogen, US). Afterwards, cDNA product was digested with NlaIII and then linked to first sequencing adapter. The product of ligation was digested with MmeI and linked to the second adapter. Then, the double adapter-flanked tags were amplified and products were purified using Spin-X filter columns. Finally, mRNA libraries were sequenced on the Illumina Genome Analyzer II (Illumina Inc, US) system following the manufacturer’s protocol. The expression profiling dataset was submitted to Gene Expression Omnibus (GEO) under the accession number of GSE47702.

### Analyses of sequencing data

Details of primary analyses of DGE sequencing data were described before [13]. In brief, all of the 17-bp DNA sequences next to the NlaIII restriction sites on human reference genome (hg19) along with the 4-bp CATG recognition site were extracted and concatenated as a new reference [14]. Tags were mapped to the constructed reference using SOAP2 allowing no more than one mismatch [15]. Normalized TPM (transcripts per million clean tags) values and fold change (absolute value of log2ratio, cancer versus normal) were calculated using uniquely mapping tags. Subsequently, candidates of differentially expressed genes were determined using the significance test described by Audic and Claverie, in which a p-value for each gene was calculated for each of the 10 cancer-normal pairs [16]. We then calculated the false discovery rate (FDR) to control the proportion of false positive results [17]. For the comparison with microarray data, we used Venny (http://bioinfogp.cnb.csic.es/tools/venny/) to generate the Venn diagram, and statistical significance of overlapping was calculated using hypergeometric test by R (http://www.R-project.org). A two-way unsupervised hierarchical clustering was done using average linkage and uncentered Pearson correlation metric by Gene Cluster 3.0, and results were visualized using TreeView [18].

For pathway enrichment, we took all the recurrently deregulated genes as input for Cytoscape with ClueGO plug-in [19, 20]. To mine out the cancer relevant genes, we performed leading edge analysis of gene set enrichment (GSEA) analysis tool [21]. Core genes ranked at the both ends on the heat map of each gene set were most significantly discrepant between tumorous and matched normal tissues. In our study, all the deregulated genes were interrogated in the gene sets of ‘Pathway in cancer’ (hsa05200) curated by KEGG and ‘Cancer molecular’ in MSigDB database [21, 22]. Besides, we performed GeneMANIA analysis to search genes that have co-expression, physical interaction, pathway relationship and shared protein domain with *ALDH2, CCNE1* and *SMAD3* [23].

### qPCR

Ten genes with wide range of fold change (-11.4 to 4.5) were selected for technical validation to check the reliability of the analytical methods for detecting differentially expressed genes with various fold changes. As described [13], we performed qPCR testing the expressions of these 10 genes in both cancer and matched normal tissues of the 10 patients in discovery screen. The expression level of each gene was normalized with U6 as it was highly expressed and stable in our samples. Value of ∆C_t_ = C_t-gene_ - C_t-U6_ was calculated for each gene. All the primers are listed in Additional file 1: Table S2.

### Immunohistochemistry scoring and survival analysis

Immunohistochemistry (IHC) assay was performed as described before [24]. Formalin-fixed paraffin-embedded (FFPE) sections after IHC staining were reviewed for the degree of immunostaining and scored by 2 independent observers based on the proportion of protein-expressing tumor cells: 0, no positive cells; 1, <5%; 2, 6%-25%; 3, 26%-50%; 4, 51%-75%; and 5, >75%. The staining intensity was graded according to the mean optical density: 0, no staining; 1, weak staining (light yellow); 2, moderate staining (yellow brown); and 3, strong staining (brown). We utilized proportion of protein-expressing cancer cells and staining intensity to calculate the staining index representing the protein expression.

We dichotomized the patient cohort based on the protein expression of ALDH2, CCNE1 and SMAD3: high-expression groups with staining index score of ≥ five and low-expression group withs score of ≤ four. Fisher’s exact test and chi-square test were performed using GraphPad Prism 6 where appropriate to test the correlation between protein expression and clinicopathologic variables. Besides, to examine the association between expression and prognosis, survival curves were estimated using the Kaplan-Meier model carried out by GraphPad Prism 6, and curves were compared using the log-rank test. We also performed multivariate (i.e. gender, age, T stage, and three molecular indicators) cox regression analysis using SPSS 21 to determine the independent prognostic factors.

## Results

### Landscape of gene expression profile of UTUC

**Figure 1:**
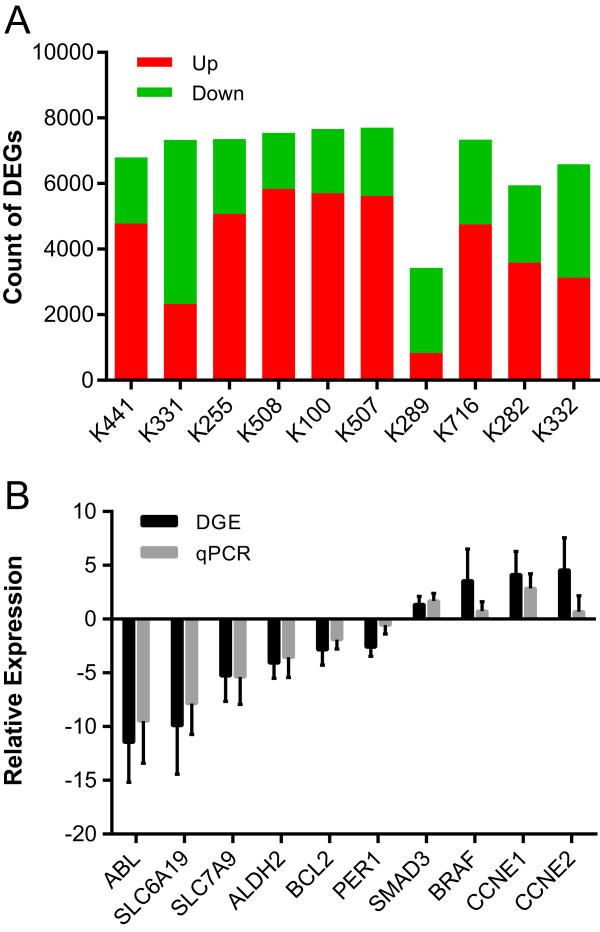
**Number of differentially expressed genes (DEGs) in each patient and qPCR validation of differential expression. A**, Number of up-regulated (red) and down-regulated (green) genes in each patient. **B**, Log2ratio determined by sequencing analysis (DGE, black) and -∆∆CT value from quantitative PCR (qPCR, grey) are shown (mean ± S.D.).

We carried out gene expression profiling using digital gene expression (DGE) sequencing in cancer and match-normal tissues of renal pelvis from 10 patients with UTUC (Additional file 1: Table S1). We first examined the numbers of genes detected under different sequencing depths, gene numbers (ranging from 15,874 to 17,546) almost saturated when the clean tag number was up to four millions (Additional file 2: Figure S1), our sequence data therefore is capable of detecting nearly all the transcribed genes in our samples. From 14,833 to 16,605 expressed genes were detected in 10 patients, and summaries of mapping results were shown in Additional file 1: Table S3. By comparing the mRNA expressions in cancer and matched normal tissues, we identified from 3431 to 7702 significantly deregulated genes (fold >1 and FDR <0.1%) across the 10 patients (Figure 1A). Besides, 5231 mRNAs were recurrently deregulated (at least five cases, and average fold >1, Additional file 1: Table S4), of which 3248 and 1983 were up- and down-regulated, respectively. Differential expression analysis using similar pipeline was validated by qPCR with the successful rate of ~88% [13]. In our study, the expression patterns of 94 of the 100 gene × patient pairs from qPCR were consistent with sequenced results (Figure 1B), which demonstrated the high reliability of our analytical pipeline.

To examine the pathway perturbations in UTUC, We subjected all the recurrently deregulated genes to ClueGO for pathway enrichment [19]. As shown in Additional file 1: Table S5, significantly disrupted pathways (corrected *P <0.05) were distributed mainly in two functional categories. Cell proliferation-related pathways (e.g. p53 signaling and cell cycling, etc.) were up-regulated. Interestingly, we also identified many metabolic pathways like PPAR signaling pathway, and Glycine serine and threonine metabolism pathways were significantly enriched with down-regulated genes, which may suggest the metabolic abnormalities in UTUC as observed in clear cell renal cell carcinoma (ccRCC) [9, 13].

### Expression profile of UTUC possesses both shared and tumor-specific molecular features compared to UC of bladder

**Figure 2:**
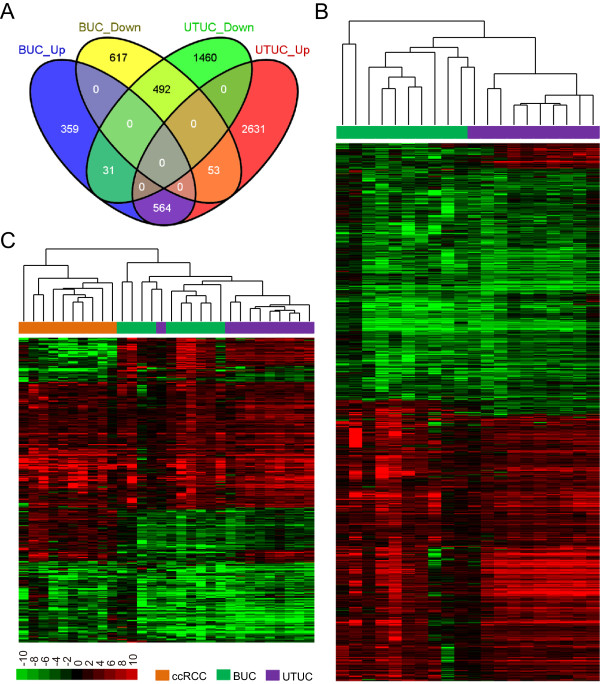
**UTUC possesses both shared and tumor-specific molecular features with bladder UC. A**, Venn diagram shows the comparison of deregulated genes in UTUC and bladder UC (BUC). The list of deregulated gene of UTUC is significantly overlapped with the deregulated genes in bladder UC, with 492 down-regulated and 564 up-regulated genes were shared. **B**, Unsupervised hierarchical clustering of 1140 genes deregulated in both UTUC and bladder UC. Both gene and sample clustering were done using average linkage and uncentered Pearson correlation metric by Cluster 3.0, and results were visualized by TreeView. Hierarchical trees of gene clustering are not shown. **C**, Unsupervised hierarchical clustering of 372 genes deregulated in UTUC, bladder UC as well as ccRCC. Analyses were done as described in **B**.

Current knowledge of UTUC is often based on the studies of bladder UC [2, 3]. However, studies have revealed that big differences exist regarding clinical behaviors and even molecular biology between the upper and the lower urinary tract urothelial carcinoma [4, 6–8]. We therefore compared the expression profile of UTUC with that of bladder UC published before [9]. We employed the same filtration criteria to detect differentially expressed genes, and found significant overlapping (***P <0.0001, hypergeometric test) between these two datasets (Figure 2A), with 492 down-regulated and 564 up-regulated genes were shared. We next interrogated the functions of genes that were share, or specific in one of the two cancers. As shown in Additional file 1: Table S5, genes commonly up- or down-regulated in UTUC and bladder UC were mainly implicated in pathways associated with cell proliferation. For instance, Cell cycle and p53 signaling pathway were the two most significant enriched pathways, and growth signal transduction pathways like MAPK and PI3K-Akt signaling pathways were significantly disrupted as well. Genes that specifically dysregulated in UTUC were associated with metabolic disorders (e.g. down-regulation of glycine, serine and threonine metabolism and PPAR signaling pathways). Genes specifically dysregulated in bladder UC were associated with adhesion related pathways (e.g. Focal adhesion and ECM-receptor interaction). These results suggested both common and tumor-specific abnormalities in UTUC and bladder UC.

We next performed hierarchical clustering with 1140 genes (Figure 2B) that were deregulated in both UTUC and bladder UC. Although one UTUC was clustered with bladder UCs, nine out of ten UTUCs were clustered together as a distinct cluster. A subset of genes were up-regulated in UTUCs but down-regulated in bladder UCs (Figure 2B, top) though these two cancers showed overall similar expression profiles. We also performed hierarchical clustering with 372 genes (Figure 2C) that were deregulated in UTUC, bladder UC as well as ccRCC [9, 13]. Interestingly, nine of ten UTUCs clustered together as a distinct subcluster as in Figure 2B, and all UTUCs were clustered with bladder UCs as a larger subcluster being separated from cluster of ccRCCs. Taken together, results shown above suggest that UTUC share significant proportion of expression profile with bladder UC, but these two cancers also characterized by tumor-specific molecular features.

### ALDH2, CCNE1 and SMAD3 are cancer relevant and associated with overall survival in patients with UTUC

**Figure 3:**
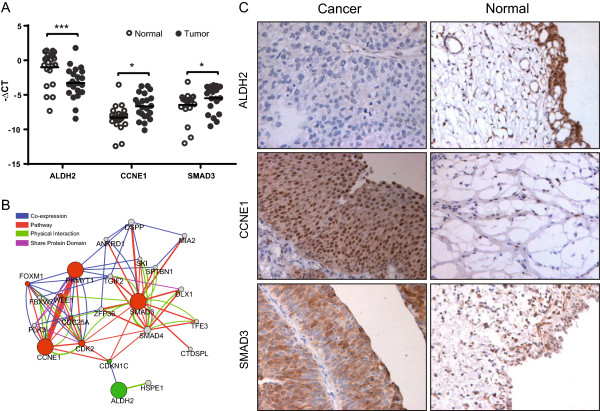
**ALDH2, CCNE1 and SMAD3 are significantly deregulated in UTUC and associated with cancer-relevant functions. A**, qPCR results of ALDH2, CCNE1 and SMAD3 in 10 patients in the discovery screen plus 12 independent cases. -∆CT value normalized with U6 is presented as mean ± S.D. to show the gene expression level in tumor and normal adjacent tissues. ***P <0.001, **P <0.01, *P <0.05, paired t-test. **B**, Network of related genes of *ALDH2, CCNE1* and *SMAD3*. Genes associated with *ALDH2*, *CCNE1* and *SMAD3* in terms of co-expression (blue lines), pathway relationship (red), physical interaction (green), or sharing protein domain (purple) are identified using GeneMANIA. The thickness of the line reflects the degree of association between two genes. The node size reflects how often paths start at the given gene node end up in one of the query genes and how long and heavily weighted those paths are. And the node colors indicate down-regulation (green) or up-regulation (red) of the genes. **C**, Representative IHC staining patterns for ALDH2, CCNE1 and SMAD3 in patients with UTUC and healthy people are shown (magnification 400X). CCNE1 and SMAD3 were strongly stained in the cancer tissues but low in the normal tissues, whereas the ALDH2 was stained low in cancer tissues.

To identify the potential cancer-relevant genes in UTUC, we performed leading edge analysis of GSEA to identify the genes that are significantly aberrant in cancer pathways and correlative with cancer molecular [21]. The resulting gene list included well-recognized tumor suppressors and oncogenes like *TP53*, *HRAS*, *PIK3CA* and *CCND1. ALDH2, CCNE1* and *SMAD3* were selected from the list for further investigations because they were implicated in the significantly disrupted pathways in UTUC. CCNE1 and SMAD3 are implicated in the regulation of cell cycling and growth signal transduction, while ALDH2 is a key player in multiple metabolic pathways. As shown in Figure 3A, their mRNA expressions were significantly different between cancer and match normal tissues of 22 patients with UTUC (***P <0.001, paired t-tests). We further interrogated the functions of *ALDH2, CCNE1* and *SMAD3* by searching genes that are functionally similar, or have shared properties using GeneMANIA [23]. As shown in Figure 3B, Genes associated these three genes were significantly enriched in G1/S (q-value <0.0001) and G2/M (q-value <0.0001) transition of mitosis, and regulation of TGF-β signaling pathway (q-value <0.001). *SMAD3*, a part of TGF-β signaling, interacted with other members (e.g. *SMAD4*, *SKI* and *CDKN1C*) in this pathway. On the other hand, *CCNE1* interplayed with many other genes regulating the transition of mitotic cell cycle (e.g. *CDC25A*, *CDK2* and *CDKN1C*). ALDH2 co-expressed with *CDKN1C*, which was also interplay with *SMAD3* and *CCNE1*. Taken together, the deregulation of *CCNE1*, *SMAD3* and *ALDH2* may lead to the disruptions of cellular functions of cell cycle control, tumor growth and metabolism.

**Figure 4:**
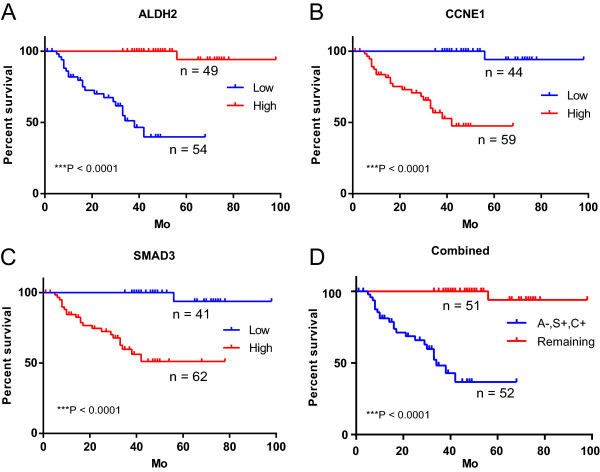
**ALDH2, CCNE1 and SMAD3 are associated with overall survival in patients with UTUC. A**-**C**, We dichotomized the 103 patients based on the protein expressions, and evaluated the association of protein expression of ALDH2 **(A)**, CCNE1 **(B)** and SMAD3 **(C)** with overall survival rate using log-rank test. **D**, Different prognosis of patients with low (-) expression of ALDH2 and high (+) expression of CCNE1 and SMAD3 (A-, S+, C+), and the remaining patients were shown. Mo: month.

To examine the prognostic roles of ALDH2, CCNE1 and SMAD3, we tested their protein expressions in FFPE samples of cancer and adjacent normal tissues from 103 patients with UTUC (Additional file 1: Table S6) using IHC assay. None of the patients underwent radiotherapy or chemotherapy before surgery. As shown in Figure 3C, CCNE1 and SMAD3 were strongly stained in the tumor tissues but were weak in the normal tissues, whereas the ALDH2 staining showed the reverse pattern. We then dichotomized the 103 patients based on the protein expressions of ALDH2, CCNE1 and SMAD3 in cancer tissues (see in the methods). The correlation between protein expressions and clinicopathologic variables is shown in Table 1. Low ALDH2, high CCNE1 and SMAD3 were significantly associated with increase in tumor depth (T1, T2 and T3; P <0.05, chi-square test). Interestingly, their expressions were also significantly correlated with each other (***P <0.0001, Fisher’s exact test), low ALDH2 was associated with high CCNE1 and SMAD3. Next, we examined the prognostic values of ALDH2, CCNE1 and SMAD3 using Kaplan-Meier analysis. As shown in Figure 4A-C, low expression of ALDH2 was significantly associated with an adverse outcome, whereas high CCNE1 and SMAD3 were associated with adverse outcomes (all ***P <0.0001, log-rank test). In addition, the predictive powers of ALDH2, CCNE1 and SMAD3 alone, and the combined marker (low ALDH2, high CCNE1 and high SMAD3, Figure 4D) were similar, which may in part be explained by their significant associations with each other (Table 1). In addition, multivariate Cox-regression analysis indicated that the expressions of ALDH2, CCNE1 and SMAD3 were independent diagnostic indicators. Besides, we found that T1 patients had more favorable outcome than T3 patients (P =7.27 × 10^-6^; Hazard ratio =0.10), but we didn’t identify a significant survival difference between T2 and T3 patients (Table 2), which was also indicated by Kaplan-Meier analysis. Expressions of ALDH2, CCNE1 and SMAD3, however, were able to identify the subgroup with higher mortality risk within the patients in T2 and T3 stages (Additional file 2: Figure S2). Further studies involving more patients will be needed to confirm whether the molecular markers can outperform the TNM staging in the outcome prediction within the subgroup of patients in T2 and T3 stages.

**Table 1:** **Association between clinicopathologic variables and molecular markers**

	ALDH2	CCNE1	SMAD3	T
ALDH2				
CCNE1	<0.0001			
SMAD3	<0.0001	<0.0001		
T^a^	0.015	0.027	0.034	
Gender	0.823	0.819	0.492	0.712

^a^Association between tumor depth (T, including T1, T2 and T3 stages) and protein expression and gender was calculated with chi-square test, other p-values were calculated with Fisher’s exact test.

**Table 2:** **Cox regression analyses for determining outcome based on the expression of ALDH2, CCNE1 and SMAD3**

Variables	No. patients	Univariate		Multivariate	
		p-value ^a^	HR (95% CI)	p-value ^b^	HR (95% CI)
**Sex**					
Female	26	0.98	0.99 (0.39-2.51)	0.92	1.05 (0.40-2.73)
Male	77				
**Age**	32-87 years	0.96	1.00 (0.96-1.04)	0.97	1.00 (0.96-1.04)
**T stage**					
T1	62	7.32 × 10^-06^	0.10 (0.03-0.27)	7.27 × 10^-06^	0.09 (0.03-0.27)
T2	16	0.12	0.413 (0.14-1.26)	0.12	0.41 (0.13-1.26)
T3	25				
**ALDH2**					
Low	54	1.69 × 10^-04^	52.02 (6.63-407.94)	2.11 × 10^-04^	81.91 (7.96-842.24)
High	49				
**CCNE1**					
Low	59	4.53 × 10^-04^	41.4 (5.17-331.66)	6.38 × 10^-04^	36.27 (4.24-309.92)
High	44				
**SMAD3**					
Low	62	9.51 × 10^-04^	31.14 (4.05-239.33)	1.04 × 10^-03^	61.90 (5.80-660.64)
High	41				

^**a**^P-value and hazard ration (HR) were calculated for each variable using Cox regression model. Sex: female vs. male; Age: continuous variable and range of age is shown instead of number of patients; T stage: T1 vs. T3, T2 vs. T3; ALDH2: low vs. high; CCNE1 and SMAD3: high vs. low. ^**b**^P-value and HR of each molecular marker were adjusted for clinicopathological factors and determined separately with Cox regression model.

## Discussion

UTUC is an aggressive and heterogeneous cancer. And because of the rarity, comprehensive study on molecular basis of UTUC is rare. To our knowledge, the present study is the first exploration of genome-wide mRNA expression patterns of UTUC using massively parallel sequencing.

Current knowledge of UTUC is mainly based on the studies of bladder UC [2, 3]. However, studies have suggested that the clinical behaviors between the upper and the lower urinary tract urothelial carcinoma can be different [4–6], UTUCs have a greater tendency towards high-grade disease than bladder UCs [5, 25, 26]. Studies of molecular insights also suggested the difference of these two cancers. Catto et al. found that distinct patterns of microsatellite instability and promoter methylation of selected loci occur in these cancers [7, 8]. Izquierdo et al. examined expressions of 13 genes relevant to bladder UC in UTUC, nine of the genes showed significant deregulations while four genes showed no significant difference [27]. Moreover, none of these 13 genes were correlated either tumor progression or survival in patients with UTUC. Liang et al., however, identified that insulin-like growth factor-binding protein-5 (IGFBP-5) was highly up-regulated in both UTUC and bladder UC, and IGFBP-5 was associated with advanced tumour stage and inferior survival in both cancers. These studies together suggests that there are shared and tumor-specific features between UTUC and bladder UC. However, the conclusions by above studies may be limited by the fact that only some selected genes were examined. In the present study, we compared the genome-wide expression patterns of UTUC with those of bladder UC, and found that these two cancer share large proportion of expression profile, which are consistent with a published study investigating the expression profiles of UTUC and bladder compared to healthy individuals using microarray [28]. Using hierarchical clustering of expression profiles, the authors found that UTUCs and bladder UCs were clustered together being separated from healthy controls [28]. The authors also identified a small subset of genes that were differentially expressed between UTUC and bladder UC. In our study, we found that, compared to bladder UC, UTUC is characterized by abnormalities in metabolic pathways, which was also observed by our group in ccRCC [9, 13]. Interestingly, kidney cancer has been suggested as a metabolic disease, many kidney cancer genes like VHL, MET and TSC1/2 are involved in metabolism-related pathways [29]. Our results suggest that metabolic disorder may be an important specific feature of UTUC compared to bladder UC.

Previous surveys of molecular prognostic indicator for UTUC were usually based on some pre-selected genes, tumor stage and grade still represent the best-established prognostic indicators [3, 4]. In our study, *ALDH2, CCNE1* and *SMAD3* were selected for further investigation based on the results of global expression profiling. All these three genes were significant and independent prognostic indicators in patients with UTUC. Our data also suggested that these molecular markers may be more robust in identifying the patient subgroup with higher mortality risk than the TNM staging, which may need to be confirmed with further investigations. ALDH2 is one of the key mediators in the disrupted metabolic pathways in our study. One of its functions is to break down acetaldehyde metabolized from ethanol, inhibition of ALDH2 therefore may result in the build-up of acetaldehyde, which is a highly toxic and carcinogenic compound [30]. Downreglation of ALDH2 has also been reported in lung cancer, and ALDH2 interacting with alcohol drinking are risk factors of stomach cancer [31, 32]. Previously, prognostic markers associated with the functions of cell cycle, proliferation, differentiation, apoptosis, and cell adhesion were evaluated in UTUC [4], our results suggested that gene associated with metabolic abnormalities could also be potential targets for developing new prognostic and therapeutic approaches for patients with UTUC.

SMAD3 is a key mediator of TGF-β signaling pathway regulating tumor growth and metastasis, and overexpression of SMAD3 was also detected in prostate cancer [33]. Other signaling transduction molecular like EGFR had been suggested as prognostic indicator in patients with UTUC [4], but the present study revealed the prognostic value of SMAD3 in UTUC for the first time. CCNE1 has been reported as an independent, unfavorable prognostic indicator in breast and Non-Small Cell Lung cancer [34, 35]. This gene is important for G1-S cell cycle control, it binds to and activates the Cdk2, and then accelerates the cell enter into S phase and achieves unrestricted tumor growth [36]. Several other cell-cycle related prognostic markers like p53, SKP2 and CKS1 for UTUC have been reported [37, 38]. Interestingly, all of p53, SKP2 and CKS1 could regulate the inactivation or activation of cyclin E-Cdk2 via mediating p21/p27. CCNE1 therefore may also represent a promising prognostic marker in patients with UTUC. Nevertheless, larger and more in-depth studies will be needed to elucidate the roles of ALDH2*,* CCNE1 and SMAD3 in UTUC.

## Conclusions

We in this study examined the genome-wide expression profile of UTUC, pathway enrichment suggested that expression patterns of UTUC are characterized by abnormalities in cell proliferation, and metabolism representing a UTUC specific feature compare to bladder UC. Importantly, we, for the first time, revealed that the protein expressions of ALDH2, CCNE1 and SMAD3 were significant and independent prognostic markers for patients with UTUC, which may facilitate the clinical management of this cancer.

## Electronic supplementary material

Additional file 1: Table S1: Clinical information of patients in the discovery screen. Table S2. Primers for qPCR validation. Table S3. Summary of sequencing and genome mapping Information. Table S4. Full list of differentially expressed genes. Table S5. Results of pathway enrichment of significantly differentially expressed genes. Table S6. Clinical information of patients in the validation screen. (XLS 2 MB)

Additional file 2: Figure S1: The number of detected genes under different sequence depths. For both tumor and normal tissues in each patient, the percentage of genes in database being detected were plotted under different number of clean tag (after filtration) and unambiguous clean tag (clean tag that uniquely maps to the genome). Figure S2. Kaplan-Meier survival plot for TNM staging and molecular indicators. The cumulative survival curve of patients in the stage of T1, T2 and T3 using TNM staging indicators was shown, as well as the association between the protein expressions of ALDH2 (B), CCNE1 (C) and SMAD3 (D) and survival rate of patients in the stage of T2 and T3. (PDF 797 KB)

## Abbreviations

UTUC: Upper tract urothelial carcinoma
UC: Urothelial carcinoma.
