# Supplementary material for: Global gene expression profiling identifies ALDH2, CCNE1 and SMAD3 as potential prognostic markers in upper tract urothelial carcinoma
Source: BMC Cancer. 2014 Nov 18;14:836. doi: 10.1186/1471-2407-14-836 (PMC4242595; doi:10.1186/1471-2407-14-836)
Supplement: Supplementary file 2 — Additional file 2: Figure S1: The number of detected genes under different sequence depths. For both tumor and normal tissues in each patient, the percentage of genes in database being detected were plotted under different number of clean tag (after filtration) and unambiguous clean tag (clean tag that uniquely maps to the genome). Figure S2. Kaplan-Meier survival plot for TNM staging and molecular indicators. The cumulative survival curve of patients in the stage of T1, T2 and T3 using TNM staging indicators was shown, as well as the association between the protein expressions of ALDH2 (B), CCNE1 (C) and SMAD3 (D) and survival rate of patients in the stage of T2 and T3. (PDF 797 KB) [file 12885_2014_5011_MOESM2_ESM.pdf]

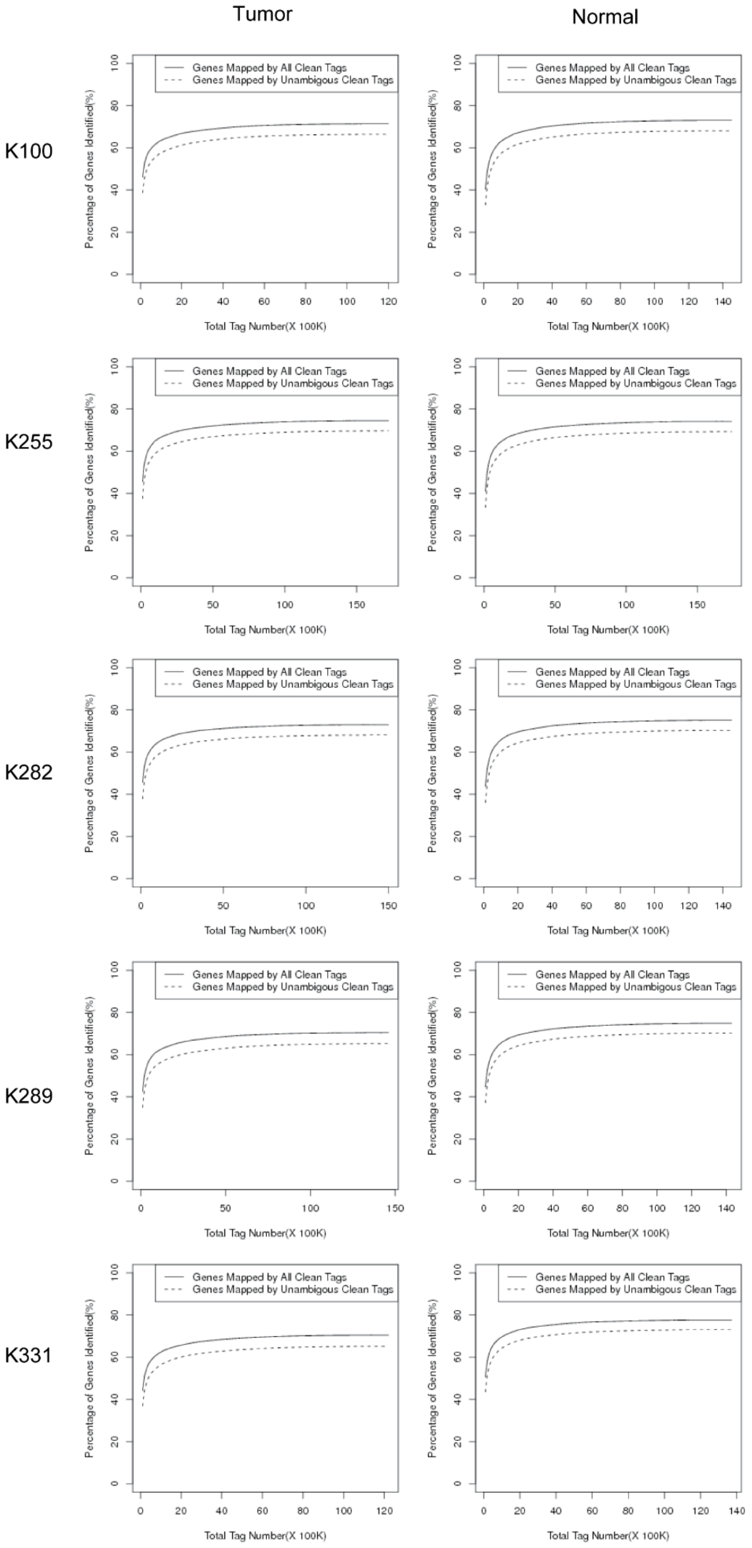
Tumor
Normal
K100
K255
K282
K289
K331
Genes Mapped by All Clean Tags
Genes Mapped by Unambigous Clean Tags
Percentage of Genes Identified(%)
Total Tag Number(X 100K)

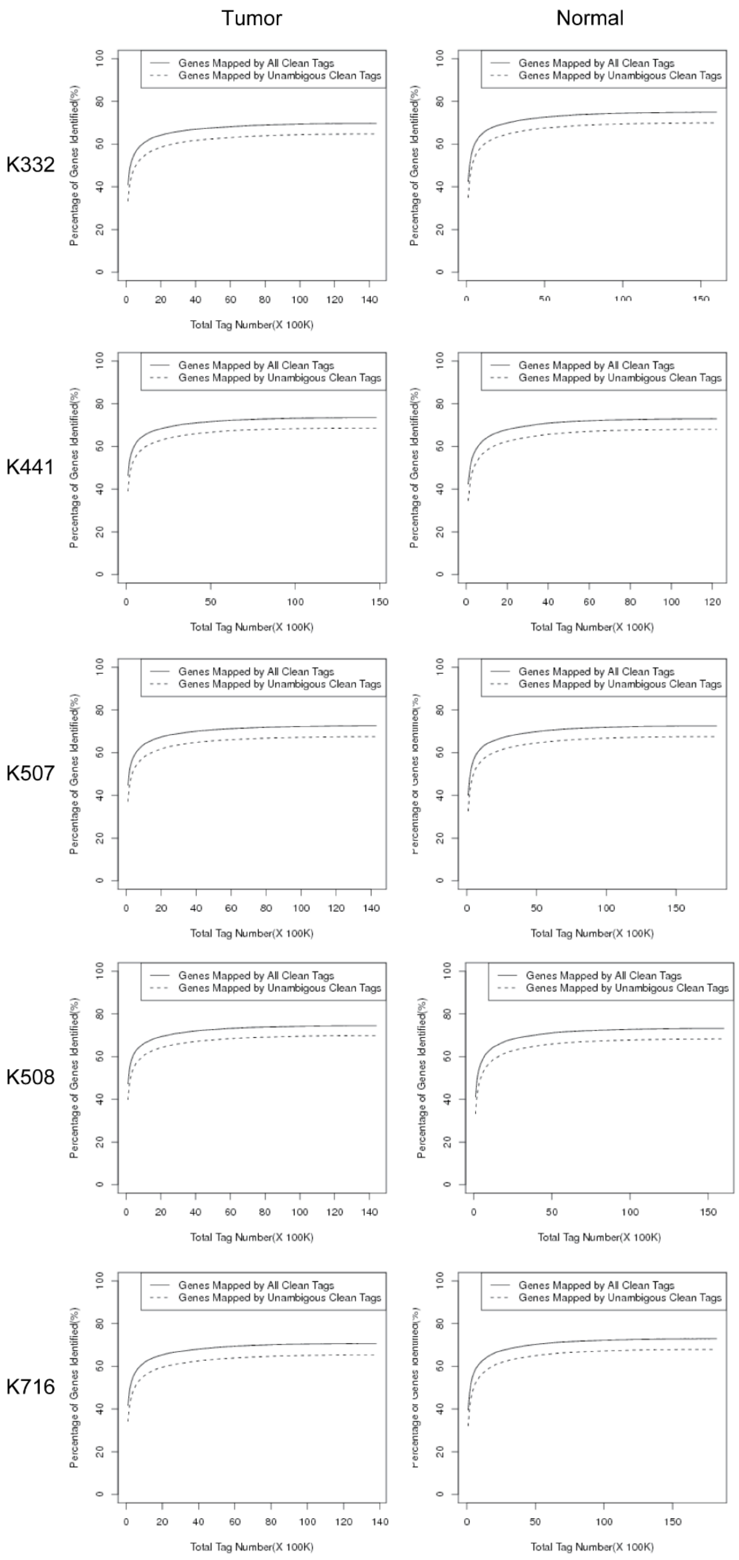

Figure S1. The number of detected genes under different sequence depths. For both tumor and normal tissues in each patient, the percentage of genes in database being detected were plotted under different number of clean tag (after filtration) and unambiguous clean tag (clean tag that uniquely maps to the genome).

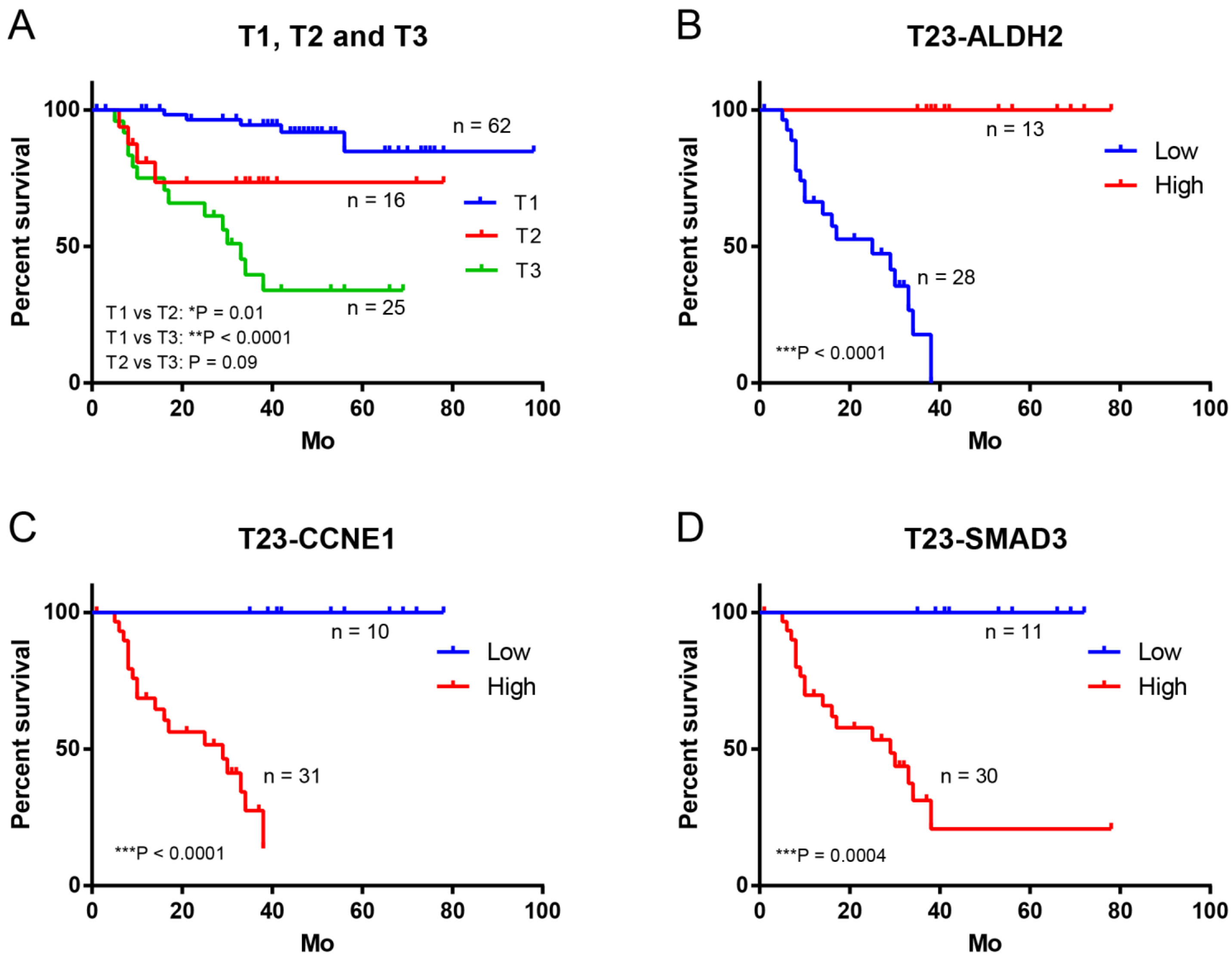

Figure S2. Kaplan-Meier survival plot for TNM staging and molecular indicators. (**A**) Cumulative survival curve of patients in the stage of T1, T2 and T3 using TNM staging indicators; (**B-D**) The association between the protein expressions of ALDH2 (B), CCNE1 (C) and SMAD3 (D) and survival rate of patients in the stage of T2 and T3. Mo: month.
